# Aberrant Expression of Glyceraldehyde-3-Phosphate Dehydrogenase (GAPDH) in Warthin Tumors

**DOI:** 10.3390/cancers12051112

**Published:** 2020-04-29

**Authors:** Robert Mandic, Abbas Agaimy, Daniel Pinto-Quintero, Katrin Roth, Afshin Teymoortash, Hans Schwarzbach, Christine G. Stoehr, Fiona R. Rodepeter, Boris A. Stuck, Michael Bette

**Affiliations:** 1 Department of Otorhinolaryngology, Head and Neck Surgery, University Hospital Marburg, Philipps-Universität Marburg, 35033 Marburg, Germany; danielpintoquintero@LIVE.COM (D.P.-Q.); prof-at@hno-zentrum-mittelhessen.de (A.T.); Boris.Stuck@uk-gm.de (B.A.S.); 2 Institute of Pathology, Friedrich-Alexander-University Erlangen-Nürnberg, University Hospital, 91504 Erlangen, Germany; Abbas.Agaimy@uk-erlangen.de (A.A.); christine.stoehr@uk-erlangen.de (C.G.S.); 3 Cellular Imaging Core Facility, Center for Tumor Biology and Immunology (ZTI), Philipps-Universität Marburg, 35043 Marburg, Germany; katrin.roth@imt.uni-marburg.de; 4 Institute of Anatomy and Cell Biology, Philipps-Universität Marburg, 35037 Marburg, Germany; hans.schwarzbach@uni-marburg.de (H.S.); bette@staff.uni-marburg.de (M.B.); 5 Institute of Pathology, University Hospital Marburg, 35043 Marburg Germany; Fiona.Rodepeter@uk-gm.de

**Keywords:** Warthin tumor, oncocyte, GAPDH, mitophagy

## Abstract

The Warthin tumor represents the second most frequent benign tumor of the parotid gland and is characterized by the presence of oncocytes rich in structurally and functionally altered mitochondria. Next to its role in metabolism, glyceraldehyde-3-phosphate dehydrogenase (GAPDH) is also implicated in cellular mitophagy. Immunohistochemistry was carried out on Warthin tumor and normal control (parotid gland with striated ducts) tissues, using anti-GAPDH specific antibodies followed by digital image analysis. Laser capture microdissection was used to isolate the oncocytic tumor cell and normal control striated duct compartments for RNA extraction and qPCR. Warthin tumor oncocytes exhibited a markedly spotted GAPDH staining pattern exhibiting cells with cytoplasmic and nuclear, only nuclear or none GAPDH staining. A significantly lower (*p* < 0.0001) total GAPDH signal was detected in Warthin tumor oncocytes. Similarly, significantly lower (*p* < 0.005) *GAPDH* mRNA levels were seen in oncocytes compared with normal ductal cells. To exclude the possibility of this GAPDH staining pattern being a general feature of oncocytic neoplasms of different organs, we tested a cohort of renal oncocytoma and oncocytic chromophobe carcinoma; none showed this type of staining. The observed progressive GAPDH loss in Warthin tumor oncocytes could be implicated in the pathogenesis of Warthin tumors.

## 1. Introduction

The Warthin tumor (synonyms: cystadenolymphoma of the parotid gland, cystadenoma lymphomatosum or cystic papillary adenoma) constitutes the second most frequent benign tumor of the salivary glands, occurring almost exclusively in the parotid gland [1]. Clinically, patients present with a slowly growing, frequently cystic mass, mainly in the inferior pole of the parotid region. Surgery represents the standard treatment procedure for this tumor. Due to its high prevalence, especially among smoking males, its frequent multifocal pattern (up to 10% bilateral) and its known tendency to undergo inflammatory complications due to its fragile cystic nature, as well as the consequences of their surgical treatment, Warthin tumors can significantly impact the patient’s quality of life. This is mainly due to occasional functional impairment of the facial nerve, which may lead to facial nerve palsy, as well as the appearance of the so-called auriculotemporal (Frey’s) syndrome after standard surgical procedures [2,3]. Known risk factors for Warthin tumor development are male gender and smoking [4]. Interestingly, previous studies observed a major increase in Warthin tumor cases [2,5,6], which appears to be, at least partly, due to a rise of smokers in the female population [6]. The authors even postulate that Warthin tumors could outpace pleomorphic adenomas, currently considered as the most-frequent tumors of the parotid gland [6]. Against this background, it is of utmost importance to develop new, alternative, non-surgical treatment strategies. For this, however, it is necessary to better understand the underlying pathomechanism leading to Warthin tumor development.

Although being described more than 100 years ago, the exact pathogenesis of this salivary gland tumor still remains ill-defined [7,8]. A leading hypothesis postulates that Warthin tumors develop from heterotopic salivary gland ductal epithelia occasionally found in parotideal lymph nodes [9,10]. Histologically, Warthin tumors are characterized by a double-layer of tall columnar oncocytes outlining variably cystic dilated glands and papillary structures embedded in a lymphocyte rich stroma. The two rows of epithelial oncocytes comprise an outer layer of columnar cells adjacent to the cystic lumen and a basal inner layer of smaller oncocytes [11]. The lymphoid stroma consists primarily of lymphocytes frequently forming germinal centers [12]. If carefully searched for, the majority of Warthin tumors showed unequivocal marginal sinuses below the tumor capsule, indicating an origin within a lymph node. This concept is further supported by frequent observations of additional multiple microscopic Warthin tumor nests within intraparotideal lymph nodes in surgical specimens of Warthin tumors. The epithelial oncocytes are considered to represent the actual “neoplastic” cell population in Warthin tumors. The origin of the oncocytes from normal salivary gland epithelia, in particular ductal cells, via metaplasia has been postulated by several groups [13,14,15]. Typically, oncocytes are large epithelial cells with a distinct eosinophilic, fine granular cytoplasm and a central small pyknotic nucleus [11]. They contain elevated numbers of mitochondria and enzymes, as seen by electron-microscopic and histochemical studies [16,17]. Accordingly, the cytoplasmic granularity and eosinophilia are ascribed to the accumulation of altered mitochondria that may occupy up to 60% of the cytoplasm [18,19]. 

Since Warthin tumor oncocytes typically harbor elevated numbers of aged mitochondria, possibly due to a reduction of cellular mitophagy, a mitochondrial dysfunction appears implicated in Warthin tumor development. Accumulation of mitochondria in the cytoplasm has been found to be associated with a common 4977-bp deletion in the mitochondrial genome [20]. 

Genomic alterations in mitochondrial or nuclear genes of oncocytes could be one explanation for a reduction in cellular mitophagy. However, there are several pathways by which mitophagy can be induced in mammalian cells [21,22,23].

Interestingly, catalytically inactive glyceraldehyde-3-phosphate dehydrogenase (GAPDH) has been found to associate with damaged mitochondria, resulting in direct engulfment of these mitochondria into lysosomes [24]. This process, also termed micromitophagy, occurs independently of the catalytic/metabolic activity of GAPDH [25] and emphasizes its many functions independent of its well-known role in cellular metabolism [26]. Remarkably, a preliminary Western blot analysis using crude lysates of presumed Warthin tumors and normal parotid gland tissues exhibited strikingly different GAPDH expression levels that could not be explained by differences in protein loading only [27]. Based on these initial observations, the present study aimed to evaluate the potential role of GAPDH in the pathogenesis of Warthin tumors.

## 2. Results

### 2.1. Validation of Warthin Tumor and Normal Parotid Gland Tissues

**Figure 1:**
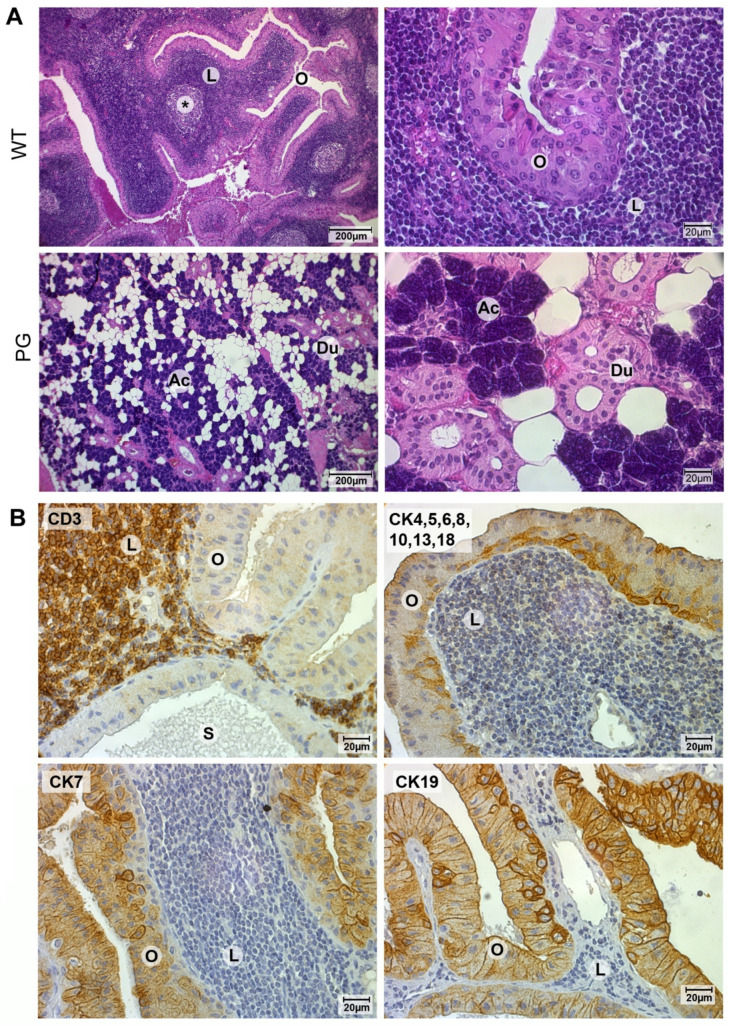
Typical histomorphological features of Warthin tumors. (**A**) Hematoxylin-and-eosin staining of Warthin tumor (WT) tissues reveals the two major cellular components, stromal lymphocytes (L) forming germinal centers (*) and epithelial oncocytes (O). The major cellular structures seen in normal parotid gland (PG) tissues are acini (Ac) and ducts (Du). (**B**) Immunohistochemical analysis using lymphocyte (CD3) and several epithelia (CK) targeting antibodies specifically mark lymphocytes (L) and oncocytes (O), respectively. S = secretion.

All tested samples were initially validated by histochemistry, using hematoxylin and eosin staining. Histologically, Warthin tumors exhibit the typical lymphocyte-rich stroma surrounded by a layer of double-rowed oncocytes, whereas normal parotid gland tissue is characterized by the presence of acini and striated ducts, with the latter representing the morphological counterpart to Warthin tumor oncocytes (Figure 1A). To further demonstrate the specific architecture of Warthin tumors, the lymphocytic compartment was highlighted by CD3 staining, whereas the epithelial oncocytic compartment was depicted by using a range of antibodies directed against cytokeratins previously found expressed in Warthin tumor oncocytes (Figure 1B) [28]. In addition, for demonstration purposes only, tumor tissue was transferred into a single cell suspension by enzymatic digestion, followed by FACS sorting to yield cell populations of the oncocytic and lymphocytic compartments, as confirmed by subsequent flow cytometry and Western blot analysis (Figure S1).

### 2.2. GAPDH Immunohistochemistry

**Figure 2:**
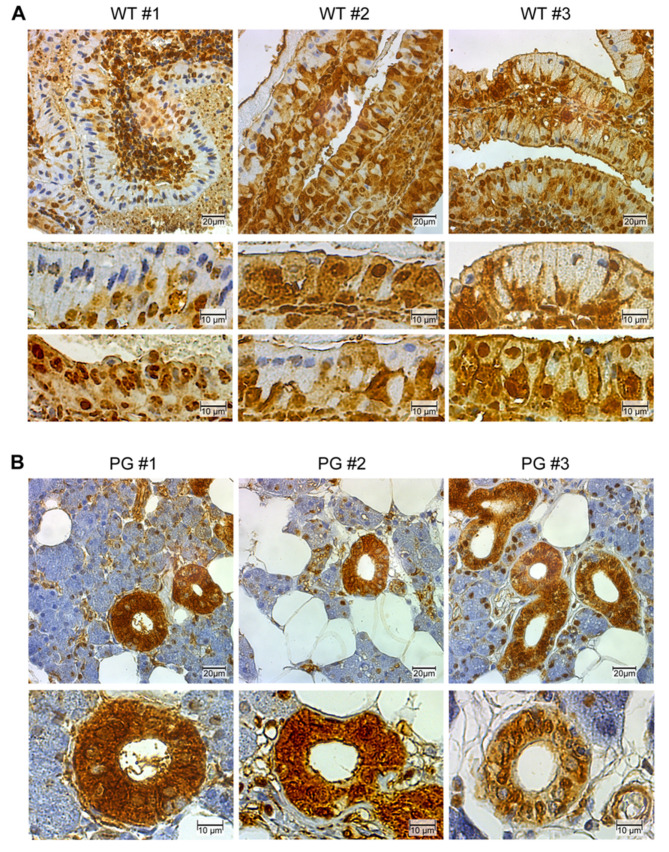
GAPDH expression in Warthin tumors and normal parotid glands. Shown are representative GAPDH immunostainings of (**A**) Warthin tumors (WT #1–3) and (**B**) normal parotid glands (PG #1–3). Note the strong and mostly homogeneous distribution of GAPDH in the cytoplasm and nuclei of ductal cells in normal PG and the heterogeneous “spotted” staining pattern in Warthin tumor oncocytes. Three major phenotypes can be seen in Warthin tumor oncocytes; cells with cytoplasmic and nuclear GAPDH; cells with only nuclear GAPDH; and GAPDH negative cells (mouse anti GAPDH, clone 0411).

Immunohistochemical analysis of Warthin tumor and normal salivary gland tissues, using a GAPDH specific antibody (clone 0411), revealed a highly distinctive “spotted” GAPDH phenotype (Figure 2A), whereas normal oncocytic ductal cells exhibited a more uniform staining pattern (Figure 2B). In particular, the spotted appearance of Warthin tumor oncocytes could be ascribed to three different GAPDH phenotypes. One phenotype, more frequently seen on basal oncocytes, exhibits a high GAPDH cytoplasmic and nuclear staining pattern, similarly to normal ductal cells, whereas neighboring more-outer oncocytes lose their cytoplasmic GAPDH signal, thereby either retaining nuclear GAPDH reactivity only or even becoming completely GAPDH negative (Figure 2A). A similar GAPDH staining pattern was observed with another GAPDH antibody (clone mAbcam 9484) [29].

### 2.3. Quantification of GAPDH Protein Expression

**Figure 3:**
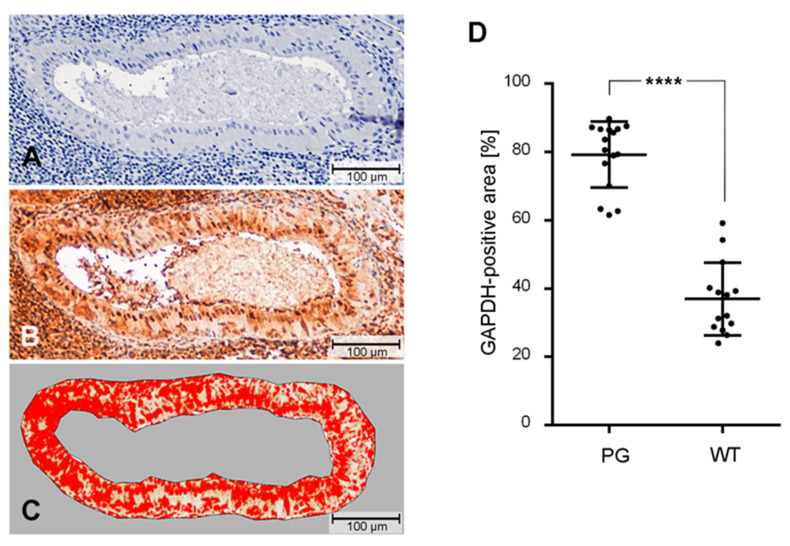
Evaluation of GAPDH immunoreactivity in ductal cells of normal parotid glands and Warthin tumor oncocytes. Tissue slices of parotid glands (PG) or Warthin tumors (WT) (**A**) were immunostained with a GAPDH specific antibody (**B**), slices were digitized and the GAPDH positive (red) area was analyzed with the program ImageJ/Fiji to receive the percentage of GAPDH positive oncocytes or ductal cells (**C**) [30]. Statistical differences between the two groups were calculated by a two-tailed unpaired t-test with Welch’s correction. (**D**) Box plot of *n* = 16 PG and *n* = 14 WT. **** *p* < 0.0001.

Digital image analysis of anti GAPDH stained Warthin tumor (*n* = 14) and normal parotid gland (*n* = 16) tissues was performed to assess the relative level of GAPDH positive Warthin tumor oncocytes in comparison with normal ductal cells (Figure 3). This analysis confirmed that Warthin tumor oncocytes exhibit a significantly lower (*p* < 0.0001) percentage of GAPDH positive cells compared to normal parotid gland ductal cells (Figure 3). 

### 2.4. Quantification of GAPDH mRNA Expression

**Figure 4:**
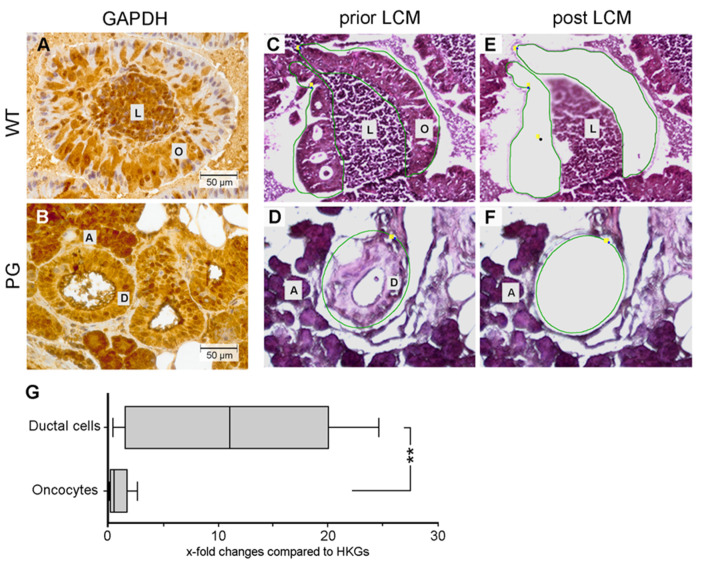
Evaluation of *GAPDH* mRNA levels in Warthin tumor oncocytes and normal ductal cells of the parotid gland. Representative GAPDH staining of Warthin tumor (WT) oncocytes (**A**) and normal parotid gland (PG) ductal cells (**B**). Areas of WT oncocytes (**C**) or PG normal ductal cells (**D**) were encircled (green lines) and captured by laser micro dissection (**E,F**). (**G**) Expression of *GAPDH* mRNA in comparison to the mean mRNA expression of the housekeeping genes (HKG) *RPLP0* and *RPL32*. Box-plot depiction of *n* = 8 PG and *n* = 8 WT samples. Statistical significance: ** *p* < 0.005. L = lymphocytes, O = oncocytes, A = acini, D = ductal cells.

Since reduced GAPDH protein expression, as seen during immunohistochemistry, does not necessarily correlate with *GAPDH* mRNA expression levels, quantitative RT-PCR was performed to assess *GAPDH* transcript levels present in Warthin tumor oncocytes and normal ductal cells. For this, the target structures (oncocytes and normal ductal cells, Figure 4A,B) were obtained by laser-capture microdissection, as depicted in Materials and Methods (Section 4) and shown in Figure 4C–F. Similarly, as observed for GAPDH protein levels after digital image analysis (Figure 3), *GAPDH* mRNA levels also appeared significantly reduced (*p* < 0.005) in Warthin tumor oncocytes, compared to normal ductal cells (Figure 4G).

### 2.5. Control Renal Tumor Cohort

None of the renal oncocytomas and oncocytoid chromophobe renal cell carcinomas displayed the aberrant pattern of GAPDH staining as described above for salivary gland Warthin tumors [31].

## 3. Discussion

Glyceraldehyde 3-phosphate dehydrogenase (GAPDH) is an enzyme that is well-known for its role in metabolism. The *GAPDH* gene is located in the short arm of chromosome 12 (12p13.31), and its eponymous metabolic function relates to the breakdown of glyceraldehyde 3-phosphate to D-glycerate 1,3-bisphosphate, a central step during glycolysis. Tumors are known to highly depend on the so-called Warburg effect, also known as aerobe glycolysis, where GAPDH is the rate-limiting enzyme [32]. How reduction or loss of GAPDH in Warthin tumor oncocytes affects tumor cell metabolism is unclear and requires further studies. Interestingly, apart from its role in metabolism, GAPDH is also implicated in diverse non-metabolic functions such as cell survival, apoptotic cell death and transcription [26]. The present study could demonstrate a characteristic immunohistochemical GAPDH staining pattern in Warthin tumor oncocytes pointing to a progressive loss of cytoplasmic GAPDH which appeared to be due to total cellular loss or nuclear shift of the protein.

One of the characteristic ultrastructural features of oncocytes is their high content of mitochondria that appear morphologically different from normal mitochondria, as observed previously [33]. Furthermore, these mitochondria also turn out to be functionally compromised, possibly due to metabolic disturbances, as reported by Li and colleagues [34]. In addition, genetic changes, such as deletions in the mitochondrial genome, were implicated in the pathogenesis of Warthin tumor development, too. Here, the authors discuss cigarette smoking, an epidemiologically known risk factor for Warthin tumor development, leading to high intracellular oxidative stress, as an underlying cause for the observed genetic changes [4,35]. Similar mitochondrial changes were also seen in renal oncocytomas [36]. In this context, it needs to be noted that a small subset of Warthin tumors, similar to mucoepidermoid carcinoma, exhibit CRTC1/MAML2 fusion transcripts due to a t(11;19) translocation [37,38].

Since mitochondrial accumulation is a typical feature of oncocytes, it is assumed that cellular mitophagy, required for maintenance of normal mitochondria numbers, is impaired. Against this background, it is very intriguing to note that GAPDH, apart from its widely and best-known function in cellular metabolism, also plays an essential role in mitophagy. Using an oxidative stress model system, Yogalingam and coworkers demonstrated that GAPDH inactivation after protein kinase C delta (PKCδ)-mediated phosphorylation of the cytoplasmic protein leads to an impairment in cellular mitophagy, which could be reversed either by PKCδ inhibition or application of a phosphorylation defective GAPDH mutant protein [25]. Similarly, mitophagy in Warthin tumor oncocytes could be impaired solely by a reduction of cytoplasmic GAPDH levels rather than due to PKCδ-mediated phosphorylation; however, a role of PKCδ mediated GAPDH inactivation in Warthin tumor oncocytes cannot be excluded. It also should be noted that the observed GAPDH loss in Warthin tumor oncocytes demonstrated by immunohistochemistry could be due to phosphorylation of GAPDH and modification of the antibody-binding site. Although possible, this appears less likely, since, firstly, different GAPDH antibodies showed comparable staining results after immunohistochemistry, and secondly, GAPDH reduction in Warthin tumor oncocytes was noted not only on the protein but also on the transcript level. It therefore appears possible that GAPDH loss in Warthin tumor oncocytes affects regular mitophagy, leading to the typical accumulation of aged, dysfunctional mitochondria. However, specific studies focusing on the role of GAPDH in Warthin tumor mitophagy will be necessary to clear up this assumption. 

It is remarkable that GAPDH can also be found in the cell nucleus [39,40,41]. Interestingly, Nakagawa et al. proposed that GAPDH plays a crucial role in nuclear assembly, particularly the membrane fusion step, in a xenopus egg cell system [42]. However, GAPDH reportedly also acts as a transcriptional coactivator by transactivating the androgen receptor [43]. Furthermore, it is astonishing that GAPDH can have a major influence on the cell cycle. In a study by Carujo et al., the authors observed GAPDH to interact with the protein SET and to reverse the inhibition of SET on cyclin B/cdk1 by binding cyclin B and thereby promoting cell-cycle progression [44]. Ventura and colleagues described the requirement of acetylation at amino acid positions 117, 227 and 251 in lysine residues of human GAPDH, for its nuclear translocation [39]. Notably, nuclear GAPDH is also intimately associated with apoptosis induction. In this context Huang et al. could demonstrate Akt2-kinase-dependent phosphorylation of GAPDH at threonine 237, to abolish its nuclear translocation and pro-apoptotic function [40]. Furthermore, Thangima Zannat and colleagues demonstrated that loss of cytoplasmic poly-(A)-binding protein results in nuclear translocation of GAPDH and posttranslational modification of p53 with subsequent induction of Bax-mediated apoptosis [45]. Against this background, the observed three GAPDH phenotypes in Warthin tumor oncocytes (cytoplasmic and nuclear, only nuclear or no GAPDH staining) demonstrated by immunohistochemistry could point to changes in its functional spectrum. 

Recent studies have brought some insight into the pathogenesis of cellular phenotypes found in neoplasms with distinctive granular eosinophilic cytoplasmic properties. Pareja et al. reported loss-of-function somatic mutations in the endosomal pH regulators ATP6AP1 and ATP6AP2 in the majority of granular cell tumors, another entity with prominent granular eosinophilic (oncocyte-like) cytoplasm [46]. Silencing of ATP6AP1 and ATP6AP2 in vitro resulted in vesicle acidification impairment, endosomal compartmental redistribution and intracytoplasmic granule accumulation that closely mimicked the granular cell tumor phenotype. To explore the possibility that GAPDH dysfunction represents a universal feature of oncocytic lesions, irrespective of their origin, we tested a control cohort of renal epithelial neoplasms with prominent oncocytic features (oncocytoma and oncocytoid chromophobe carcinoma); none showed the distinctive GAPDH phenotypes we described herein for salivary gland Warthin tumors, indicating that this expression pattern is likely specific for Warthin tumors. However, this needs verification in larger future studies encompassing oncocytic lesions from different organs.

In addition to their high prevalence in the population (being the second most-common parotid neoplasm after pleomorphic adenoma, or even in some unbiased series as common as pleomorphic adenoma), Warthin tumors are known for their frequent multifocal and bilateral appearance. This necessitates, in many cases, total parotidectomy, to avoid the need for repeated surgery and hence scarifying the facial nerve with significant impact on the patient’s quality of life. Thus, better understanding of the pathogenic mechanisms involved in Warthin tumorigenesis might pave the way for potential organ-preserving novel therapeutic targets. If GAPDH is indeed determined to be involved or even required for Warthin tumor development, then GAPDH could represent a therapeutic target that potentially could influence the course of Warthin tumor disease.

## 4. Materials and Methods

### 4.1. Tissues

Tissue samples were derived from surgically excised Warthin tumors, at the Department of Otorhinolaryngology, Head and Neck Surgery, University Hospital Marburg, Marburg, Germany, and were used according to the requirements and guidelines of the local ethics committee (ethic code: 65/15; Ethics Committee, Department of Medicine, Philipps-Universität Marburg) and in cooperation with the Comprehensive Biomaterial Bank Marburg (CBBMR, Department of Medicine, Philipps-Universität Marburg, Germany). Tissue samples were fixed in 4% buffered formaldehyde solution and embedded routinely in paraffin (FFPE). Part of the tissue samples were fresh frozen in liquid nitrogen. As a further control cohort for GAPDH immunohistochemistry, tissue microarray slides containing punches from 20 renal oncocytomas, 24 chromophobe renal cell carcinomas and one hybrid oncocytoma-chromophobe renal cell carcinoma were used. Samples were deployed in accordance with ethical guidelines for the use of retrospective tissue samples provided by the local ethics committee of the Friedrich-Alexander University Erlangen-Nuremberg (ethics committee statements 24 January 2005 (no number), 13 February 2008 (ethic code: 3755), 18 January 2012 (ethic code: 4607) and 17 November 2016 (ethic code: 329_16 B)).

### 4.2. Immunohistochemistry

Tissue slices that were 3 µm thick were generated from FFPE samples, using a microtome, and mounted onto standard glass slides, for microscopic examination. Immunohistochemical analysis was performed as previously reported, but with some modifications [47]. In short, sample-carrying microscope slides were incubated in limonene (Roti^®^-Histol, Carl Roth GmbH + Co. KG, Karlsruhe, Germany), for sample deparaffinization. After removal of limonene, using a descending alcohol gradient and rehydration, endogenous peroxidases were blocked by application of 3% (v/v) H_2_O_2_. Samples were subsequently boiled for 20 min in sodium citrate buffer (pH 6.0), for antigen retrieval. Blocking of unspecific binding sites was performed by applying normal goat serum (1:10 in 0.15 mol/L PBS) for 30 min at room temperature (RT). Subsequently, the samples were incubated with the respective primary antibodies, overnight, at 4 °C (all antibodies except for CD3 (rabbit polyclonal) were mouse monoclonal: GAPDH, clone mAbcam 9484, cat# ab9484, Abcam (Berlin, Germany); GAPDH, clone 0411, cat# sc-47724, Santa Cruz Biotechnology Inc. (Dallas, TX, USA); cytokeratin 7, clone OV-TL12/30, cat# MON3014, Monosan (Uden, The Netherlands); cytokeratin 19, clone A53-B/A2.26, cat# sc-6278, Santa Cruz Biotechnology Inc.; pan-cytokeratin, clone C11, cat# sc-8018, Santa Cruz Biotechnology Inc.; CD3, cat# A0452, Dako (North America Inc., Carpinteria, CA, USA) (except for ab9484 (1:2000) all antibodies were used at a 1:100 dilution in Dako Antibody Diluent). As a negative control, normal mouse (cat# X0931, Dako) or rabbit (cat# X0903) IgG was used at the same concentration as the primary antibodies. After rinsing the samples in PBS, EnVision+/HRP, Dual Link Rabbit/Mouse, HRP Polymer (cat# K4061, Dako) was applied for 1 h at RT. After another round of washing in PBS, Liquid DAB (Diaminobenzidine) + Substrate Chromogen System (Dako) was applied for 15 min, followed by a 3 × 5 min washing step in H_2_O and counterstaining with hemalaun (Merck, Darmstadt, Germany) for 1 min at RT. Then microscope slides were washed for 2 min in H_2_O, followed by dehydration in an ascending ethanol gradient. Corbit balsam (Fa. Hecht, Kiel, Germany) was applied, and samples were sealed with a cover slip.

### 4.3. Cell Sorting, Flow Cytometry and Western Blot Analysis

For the purpose of demonstrating and characterizing the two distinct cellular compartments present in Warthin tumors, tumor tissue (fresh frozen in DMEM/20%FCS) was dissociated with dispase and collagenase and filtered (50 µm). Cell sorting was performed on a MoFlo Astrios System (Beckman Coulter Life Sciences, Indianapolis, IN, USA) yielding a presumed lymphocyte and oncocyte cell population to be used for further validation in subsequent flow cytometry and Western blot analyses. Flow cytometry on the presumed two cell populations was performed with antibodies directed against cytokeratins (CK7, CK19, broad-spectrum CK) as markers for the oncocytic epithelia and an antibody directed against the T-lymphocyte marker CD3. Western blot analysis using the same anti CK and CD3 antibodies was performed according to a previously described protocol [48].

### 4.4. Laser-Capture Microdissection (LCM)

Six µm thick FFPE tissue slices were generated with a microtome under RNase-free conditions. Tissue slices were positioned on top of microscope slides specifically designed for LCM (Carl Zeiss™ Membrane Slides, 1.0 PEN (D) PEN (polyethylene naphthalate) membrane-covered, cat# 415190-9041-000, Carl Zeiss Microscopy GmbH, Jena, Germany). Tissue slides were dried for 1 h at 37 °C and then deparaffinized in limonene (5 min, RT) and exposed to a descending ethanol gradient (100%: 2 min, 90%: 2 min, 70%: 2 min; all at RT), followed by incubation with hemalaun (1 min, RT), wash in H_2_O (3 min, RT) and incubation with eosin for 2 min at RT. After a short rinse in H_2_O, slides were exposed to an ascending ethanol gradient (70%: 5 s, 90%: 60 s; 100%: 2 min; all at RT). After drying by air, slides were kept protected in a 50 mL tube (Falcon) at −20 °C, until LCM. LCM was carried out by using the PALM MicroBeam System (Carl Zeiss Microscopy GmbH) at the Cellular Imaging Core Facility (Faculty of Medicine, Philipps-Universität, Marburg, Germany). After microscopic visualization of the target cell regions (oncocytes, ductal cells) using the PALM RoboSoftware (Carl Zeiss Microscopy GmbH), the respective cell clusters were defined by drawing a line, thereby encircling the region of interest to be cut out by the laser. By activating the laser, the designated cell clusters were cut out and catapulted into the adhesive lid of a 500 µL reaction tube (AdhesiveCap 500 clear, cat# 415190-9211-000, Carl Zeiss Microscopy GmbH). Tubes were kept at −20 °C, until further use.

### 4.5. Nucleic Acid Extraction from Cells Isolated by LCM

The RNeasy FFPE kit (cat# 73504, Qiagen, Hilden, Germany) was used for RNA extraction out of laser-captured cells, according to a modified protocol of the supplier. In short, 150 µL of buffer PKD was added to the tube, followed by an addition of proteinase K. Tubes were incubated overnight (modification to the original protocol) at 56 °C, upside-down, followed by a heating step for 15 min at 80 °C, to reverse formaldehyde crosslinks. This step was followed by DNase digestion for 15 min at RT. All following steps were made according to the manufacturer’s protocol. The RNA yield and quality were evaluated with the Qubit (Qubit™ RNA HS Assay Kit, Thermo Fisher Scientific, Darmstadt, Germany) and Experion (Experion^TM^ RNA HighSens Analysis Kit, Bio-Rad Laboratories GmbH, Munich, Germany) Systems. The RQI (RNA Quality Indicator) of the samples was regularly found to be low (~2), which is typical for RNA extracted from FFPE tissues. This fragmented RNA, however, is suitable for downstream applications, such as quantitative PCR.

### 4.6. Digital Image Analysis with ImageJ/Fiji

Microscopic slides of GAPDH stained Warthin tumors, and normal parotid gland tissue slices were captured by digital photography, at a 20× magnification, and saved as a TIF image. Subsequently, digital image analysis was performed with the open-source program ImageJ/Fiji [30]. As a first step, the resolution of the respective microscopic image was determined, and it corresponded to 1600 × 1200 pixel in all tested images. In the next step, the region of interest (oncocytes or normal ductal cells) was encircled, yielding the actual surface to be considered during analysis. By using the Color Threshold tool of the program, the threshold was set to first capture the total surface of oncocytes or normal ductal cells, respectively. In a subsequent step, the threshold was set to only capture the GAPDH signal (DAB positive, brown precipitate). By comparing both values (GAPDH positive surface divided by total surface), the percentage of GAPDH positive oncocytes or normal ductal cells could be determined. A total of 100 images from Warthin tumor and parotid gland samples were analyzed. For each tested parotid gland sample, images were evaluated until reaching a cumulative pixel number of approximately 200,000 (the highest variation to this set number was 7.7%). Since oncocytes were more abundant in the Warthin tumor than normal ductal cells in parotid gland tissues, the Warthin tumor sample images were evaluated until reaching a cumulative pixel number of approximately 1,500,000 (the highest variation to this set number was 6.7%).

### 4.7. Quantitative RT-PCR

Primers used (all from Thermo Fisher Scientific GmbH, Dreieich, Germany) were as follows: *GAPDH1_forward*: *ACAACTTTGGTATCGTGGAAGG*, *GAPDH1_reverse*: *GCCATCACGCCACAGTTTC*; *GAPDH2_forward*: *AGCCCAGAACACTGGTCTC*, *GAPDH2_reverse*: *ACTCAGGATTTCAATGGTGCC*; housekeeping genes (HKG) RPLP0_forward: *AGCCCAGAACACTGGTCTC*, *RPLP0_reverse*: *ACTCAGGATTTCAATGGTGCC*; *RPL32_forward*: *GCCCAAGATCGTCAAAAAGAGA*, *RPL32_reverse*: *TCCGCCAGTTACGCTTAATTT*. RNA derived from LCM sample pieces was reverse transcribed with random hexamer primers, using the Transcriptor First Strand cDNA Synthesis Kit (Roche Diagnostics GmbH, Mannheim, Germany). Quantitative PCR was performed on a QuantStudio^TM^ 5 system (Thermo Fisher Scientific), using the Power SYBR Green PCR Master Mix (Thermo Fisher Scientific). The evaluation was carried out according to the ct method. The expression levels of *GAPDH* were normalized to the respective expression levels of the HKGs. For calculation, qPCR results of the two *GAPDH* primer pairs, as well as the two HKGs, were averaged.

### 4.8. Statistics

For the calculation of statistic differences between parotid glands and Warthin tumors, an unpaired, two-tailed t-test was used. For *GAPDH* mRNA expression, a Welch correction was additionally applied due to significantly different variances of the group means. In all analyses a *p*-value of <0.05 was considered to be a significant difference. The GraphPad Prism Software 6.0 (GraphPad Software Inc, San Diego, CA, USA) was used.

## 5. Conclusions

In summary, we describe herein a distinctive aberrant expression of the glyceraldehyde-3-phosphate dehydrogenase (GAPDH) in Warthin tumors. These results suggest *GAPDH* as a potential candidate gene in the pathogenesis of parotid gland Warthin tumors. How this relates to the pathogenesis of Warthin tumor development, and if this knowledge could be applied for therapeutic purposes, will have to be elucidated by further studies.

## Supplementary Materials

The following are available online at https://www.mdpi.com/2072-6694/12/5/1112/s1, Figure S1: Single cell dissociation of WT tissue allows separating the lymphocytic (Gate 1) and oncocytic (Gate 2) compartments, using FACS. Subsequent flow cytometry (A) and Western blot (B) analysis of sorted Gate 1 and Gate 2 cells, using CD3- and CK-specific antibodies validates the successful separation of both compartments.
