# Supplementary material for: Aberrant Expression of Glyceraldehyde-3-Phosphate Dehydrogenase (GAPDH) in Warthin Tumors
Source: Cancers (Basel). 2020 Apr 29;12(5):1112. doi: 10.3390/cancers12051112 (PMC7281563; doi:10.3390/cancers12051112)
Supplement: Supplementary file 1 [file cancers-12-01112-s001.pdf]

Supplementary Materials: Aberrant Expression of Glyceraldehyde-3-Phosphate Dehydrogenase (GAPDH) in Parotid Gland Warthin Tumors

Robert Mandic, Abbas Agaimy, Daniel Pinto Quintero, Katrin Roth, Afshin Teymoortash, Hans Schwarzbach, Christine G. Stoehr, Fiona R. Rodepeter, Boris A. Stuck and Michael Bette

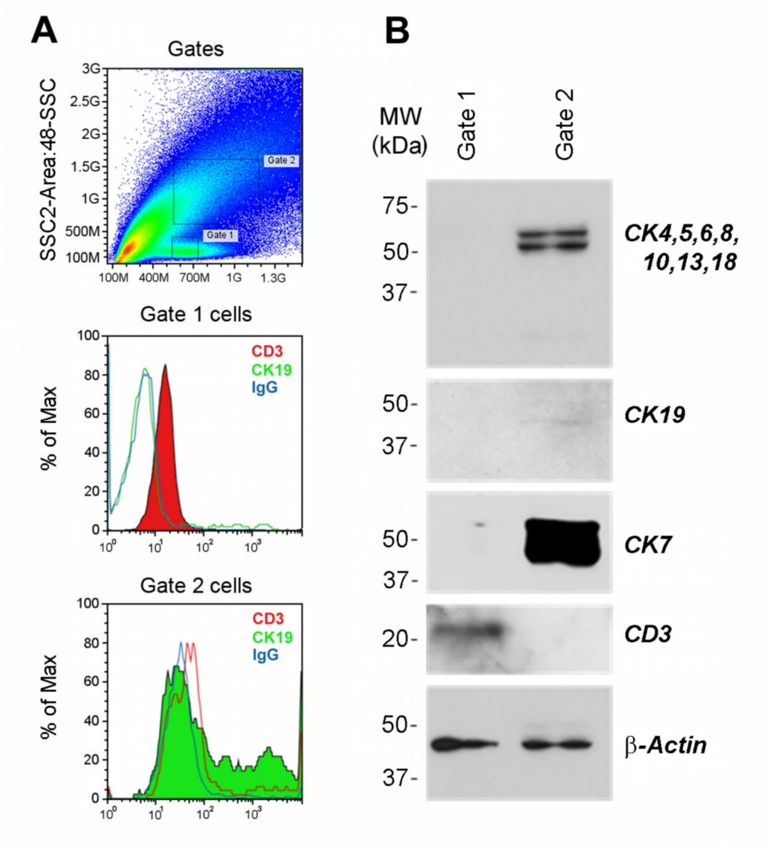

Figure S1. Single cell dissociation of WT tissue allows separating the lymphocytic (Gate 1) and oncocytic (Gate 2) compartments, using FACS. Subsequent flow cytometry (**A**) and Western blot (**B**) analysis of sorted Gate 1 and Gate 2 cells, using CD3- and CK-specific antibodies validates the successful separation of both compartments.

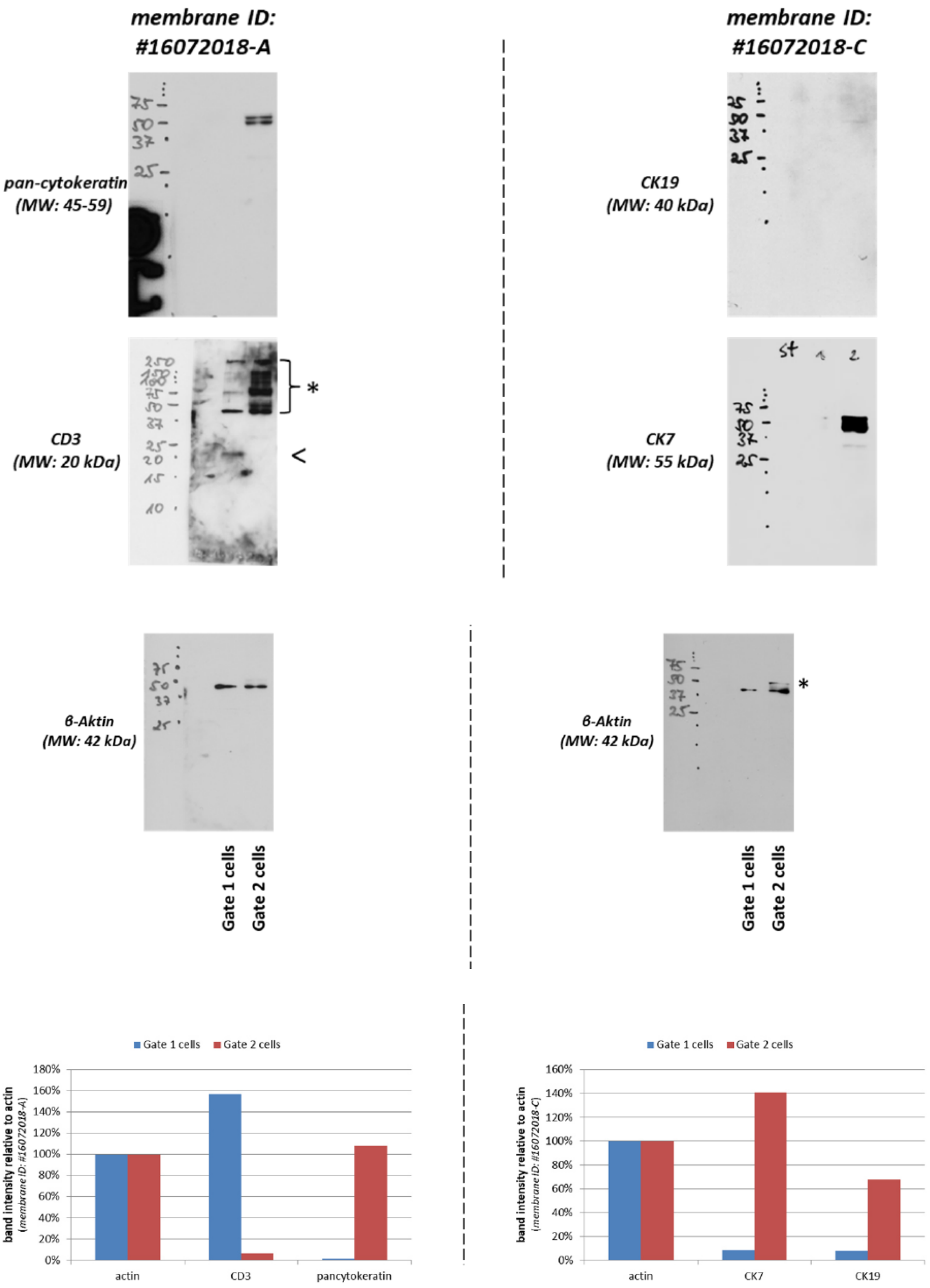

Original Western blot films showing bands of Gate 1 and Gate 2 sorted Warthin tumor cells as depicted in Figure S1. Please note that "*" refers to bands appearing after longer exposure due to the previously used antibody (pan-CK or CK7) on the same non-stripped nitrocellulose membranes. The "<" arrow refers to the CD3 band of correct size and is only seen in the Gate 1 cell lane. Shown below are quantifications of the respective bands relative to beta actin as measured with the Imagej/Fiji software. Also note: This data is derived from a single experiment and serves solely for demonstrating successfull separation of the two major compartments (epithelial oncocytes and lymphocytes) present inWarthin tumors.

© 2020 by the authors. Submitted for possible open access publication under the terms and conditions of the Creative Commons Attribution (CC BY) license (http://creativecommons.org/licenses/by/4.0/).
